# Virus–Host Interactions Drive Contrasting Bacterial Diel Dynamics in the Ocean

**DOI:** 10.34133/research.0213

**Published:** 2023-08-22

**Authors:** Xiaowei Chen, Chen Hu, Wei Wei, Yunlan Yang, Markus G. Weinbauer, Hongbo Li, Shiying Ren, Ruijie Ma, Yibin Huang, Tingwei Luo, Nianzhi Jiao, Rui Zhang

**Affiliations:** ^1^ State Key Laboratory of Marine Environmental Science, College of Ocean and Earth Sciences, Fujian Key Laboratory of Marine Carbon Sequestration, Xiamen University, Xiamen 361102, PR China.; ^2^ College of the Environment and Ecology, Xiamen University, Xiamen 361102, PR China.; ^3^ Research Center for Environmental Ecology and Engineering, School of Environmental Ecology and Biological Engineering, Wuhan Institute of Technology, Wuhan 430205, PR China.; ^^4^^ Sorbonne Universités, UPMC, Université Paris 06, CNRS, Laboratoire d’Océanographie de Villefranche (LOV), Villefranche-sur-Mer 06230, France.; ^^5^^ National Marine Environmental Monitoring Center, Ministry of Ecological Environment, Dalian 116023, PR China.; ^^6^^ Department of Ocean Sciences, University of California, Santa Cruz, CA, USA.; ^^7^^ NOAA/OAR Pacific Marine Environmental Laboratory, Seattle, WA, USA.; ^^8^^ Institute for Advanced Study, Shenzhen University, Shenzhen 518055, PR China.

## Abstract

Marine organisms perform a sea of diel rhythmicity. Planktonic diel dynamics have been shown to be driven by light, energy resources, circadian rhythms, and the coordinated coupling of photoautotrophs and heterotrophic bacterioplankton. Here, we explore the diel fluctuation of viral production and decay and their impact on the total and active bacterial community in the coastal and open seawaters of the South China Sea. The results showed that the night-production diel pattern of lytic viral production was concurrent with the lower viral decay at night, contributing to the accumulation of the viral population size during the night for surface waters. The diel variations in bacterial activity, community composition, and diversity were found highly affected by viral dynamics. This was revealed by the finding that bacterial community diversity was positively correlated to lytic viral production in the euphotic zone of the open ocean but was negatively related to lysogenic viral production in the coastal ocean. Such distinct but contrasting correlations suggest that viral life strategies can not only contribute to diversifying bacterial community but also potentially piggyback their host to dominate bacterial community, suggesting the tightly synchronized depth-dependent and habitat-specific diel patterns of virus–host interactions. It further implies that viruses serve as an ecologically important driver of bacterial diel dynamics across the ocean, highlighting the viral roles in bacterial ecological and biogeochemical processes in the ocean.

## Introduction

Biological rhythmicity, found in various living organisms from bacterioplankton to mammals, is a ubiquitous mechanism enabling organisms to adapt to recurring environmental conditions. Remarkably, the diel rhythm, which is common in photic ocean ecosystems, drives marine organisms to perform a sea of diel rhythmicity [1]. The coordination of marine biological activities into the periodic diel cycle has been well documented among phytoplankton and zooplankton at the behavioral, physiological, and molecular levels, which is shaped by exogenous and endogenous factors, including light availability, resource supply, and the circadian clock [2–5]. Moreover, recent studies have revealed the existence of diel fluctuations in multiple bacteria-mediated ocean biogeochemical processes, such as nitrogen fixation, sulfonate cycling, and triacylglycerol biosynthesis [6–8].

The diel oscillation of heterotrophic bacterioplankton, at the population-specific and community levels, has been recorded across the ocean and is generally regarded to be driven by light-dependent mechanism (e.g., proton-pumping rhodopsin) or the coordinated diel coupling of photoautotroph and heterotroph (e.g., the photosynthetic products released from the primary producer) [1,9,10]. Beyond the direct interaction and adaptation to oscillating environmental conditions such as light energy, the ocean’s spatiotemporal dynamics of the natural bacterial community are distinctly regulated by ubiquitous viral predation [11,12]. The highly active viral community implements approximately 10^23^ viral infections every second in the ocean, exerting pivotal impacts on bacterial biomass, mortality, and metabolism [13,14]. The viral attack can depress bacterial growth and activity, altering the bacterial community composition through host specificity and subsequently diversifying the bacterial community [15–17]. Over diel cycling, previous culture-based experiments and field investigations have provided certain possibilities of viral diel dynamics driven by the light–dark period or host replication and transcriptional and metabolic activities [18–21]. For example, cyanoviruses were found to reproduce viral progeny influenced by light-driven cyanobacterial photosynthetic activity during the daytime and lyse host cells at night [1,20,21]. However, whether and how virus–host interactions shape bacterial diel dynamics in the natural environment remain poorly understood.

Here, we explored the diel cycling of virus–host interactions and their impact on bacterial abundance, productivity, total and active community diversity, and composition in the coastal and open ocean water column of the South China Sea (SCS). Our results indicated that the diel pattern of the bacterial community was distinctly shaped by the depth-dependent and habitat-specific diel patterns of virus–host interactions, suggesting viral roles in driving bacterial diel dynamics in ocean ecosystems.

## Results

### Diel fluctuation of the sampling environments

**Fig. 1.**
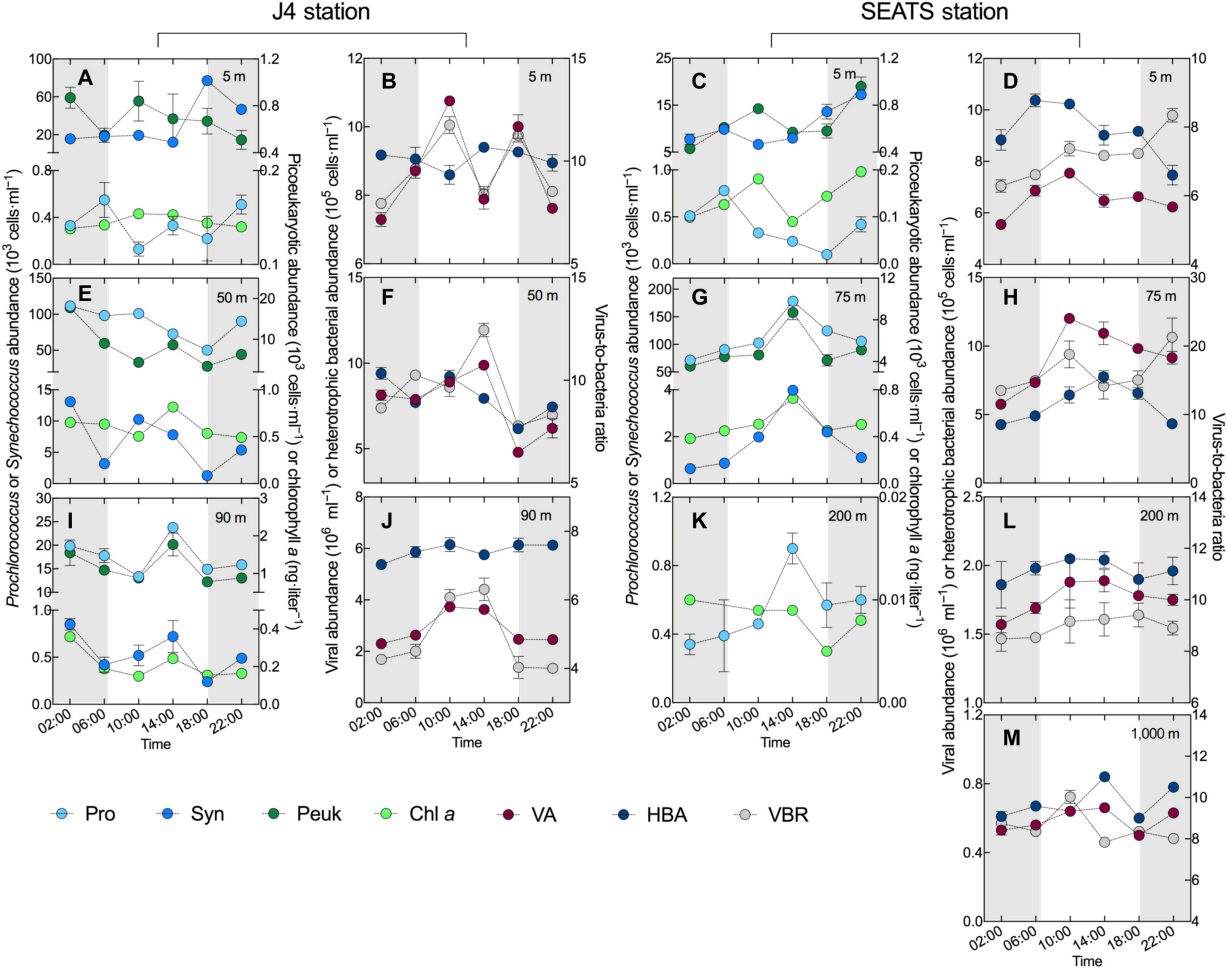
Diel variability in bacterial and viral concentrations. The abundances of *Prochlorococcus* (Pro), *Synechococcus* (Syn), and picoeukaryote (Peuk) as well as the Chl *a* concentration in the 5-m (A), 50-m (E), and 90-m (I) layers of station J4 and the 5-m (C), 75-m (G), and 200-m (K) layers of station SEATS. The VA, HBA, and the VBR in the 5-m (B), 50-m (F), and 90-m (J) layers of station J4 and the 5-m (D), 75-m (H), 200-m (L), and 1,000-m (M) layers of station SEATS. The error bars are indicated as SD, and the shaded areas represent the nighttime.

At the SCS, the largest marginal sea, samples were collected at 6 time points in a diel periodicity in different layers at coastal station J4, including the surface (5 m), deep chlorophyll maximum (DCM; 50 m), and bottom (90 m) layers, and at open ocean station for South East Asia Time-series Study (SEATS), including the surface (5 m), DCM (75 m), the boundary of the euphotic zone (boundary layer; 200 m), and mesopelagic (1,000 m) layers (Fig. S1 and Table S1). A relatively constant change in hydrologic features was observed during the sampling period at station SEATS compared to station J4 (Fig. S1), confirming the reported stable environmental conditions over the diel scale at the open ocean water column [4]. Consistent with the pattern generally found in the oceanic environment [22], peak chlorophyll *a* (Chl *a*) concentrations could be recorded during the daytime in the surface and DCM layers of J4 and SEATS, accompanied by a peak at midnight (02:00) in the bottom layer of J4 and the relatively invariant fluctuation in the boundary layer at the SEATS station (Fig. 1).

### Diel variability of bacterial abundance and activity

The abundances of photoautotrophs (i.e., *Prochlorococcus*, *Synechococcus*, and picoeukaryotes) peaked around nighttime in the surface and DCM layer of J4 and the surface layer of SEATS (Fig. 1), in a similar way as has been shown previously in a high-resolution investigation in Pacific surface water [23]. A contrasting diel pattern with the highest value at midday was found in the bottom layer of J4 and the DCM and boundary layer of SEATS. Their different diel patterns observed in the surface layer and other layers might be primarily attributed to the different activities of photoautotrophs, i.e., cell division rate, which is strongly affected by the light inhibition phenomenon during the daytime [24].

Similar to a diel cycle study in the North Sea [18], no distinct diel periodicity of heterotrophic bacterial abundance (HBA) was observed at coastal station J4. However, a pronounced diel periodicity of HBA was detectable in the surface layer of SEATS where the highest value occurred in the early morning (10.37 ± 0.25 × 10^5^ cells·ml^−1^) and then declined during the day (Fig. 1D). The HBA peaked around noon in the DCM (7.75 ± 0.46 × 10^5^ cells·ml^−1^) and boundary layer (2.05 ± 0.01 × 10^5^ cells·ml^−1^), and a similar diel periodicity was found in the mesopelagic Red Sea [4].

**Fig. 2.**
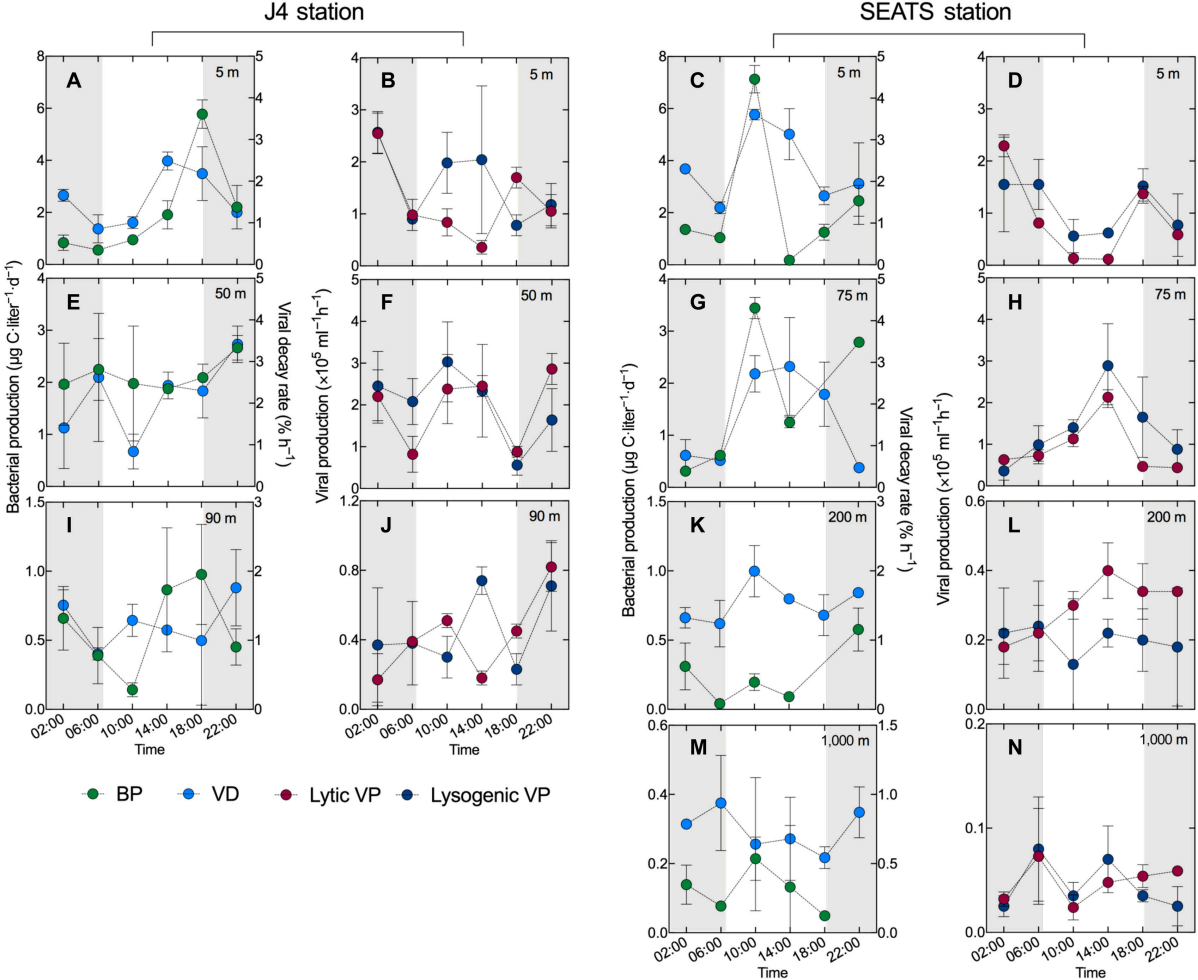
Diel variability in viral and bacterial activities. The BP and VD in the 5-m (A), 50-m (E), and 90-m (I) layers of station J4 and the 5-m (C), 75-m (G), 200-m (K), and 1,000-m (M) layers of station SEATS. The lytic and lysogenic VP in the 5-m (B), 50-m (F), and 90-m (J) layers of station J4 and the 5-m (D), 75-m (H), 200-m (L), and 1,000-m (N) layers of station SEATS. The error bars are indicated as SD, and the shaded areas represent the nighttime.

Bacterial production (BP) peaked around dusk and was lowest around dawn in all layers at J4 (Fig. 2A, E, and I). At the SEATS site, double peaks of BP were found at 10:00 (surface, 7.13 ± 0.53 μg C·liter^−1^·day^−1^; DCM, 3.45 ± 0.21 μg C·liter^−1^·day^−1^) and 22:00 (surface, 2.46 ± 0.60 μg C·liter^−1^·day^−1^; DCM, 2.79 ± 0.04 μg C·liter^−1^·day^−1^) in the surface and DCM layer (Fig. 2C and G), consistent with the diel variations reported in the Mediterranean Sea [25]. In the boundary layer, a single peak of BP (0.58 ± 0.16 μg C·liter^−1^·day^−1^) was found at 22:00, accompanied by the disappearance of the peak at 10:00 (Fig. 2K). This result showed that the magnitude of the peak during daytime (10:00) of the open ocean gradually diminished with water depth, whereas the peak during nighttime (22:00) gradually increased with depth.

### Diel variation in bacterial community diversity and composition

**Fig. 3.**
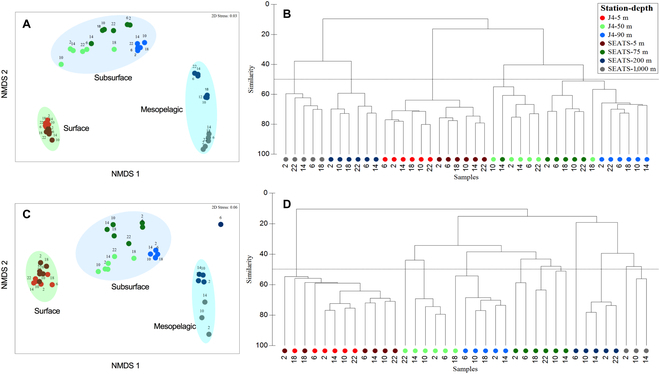
The similarity of the bacterial community composition (BCC) over diel cycling in different layers of stations J4 and SEATS. NMDS ordination with 2 dimensions between the total BCC at the DNA level (A) and active BCC at the RNA level (C). The 3 distinct groups (surface, subsurface, and mesopelagic) are represented by 3 color areas. Hierarchical clustering of Bray–Curtis similarities of the total bacterial BCC at the DNA level (B) and active BCC at the RNA level (D). The different numbers of the symbols represent the sampling time of the day.

**Fig. 4.**
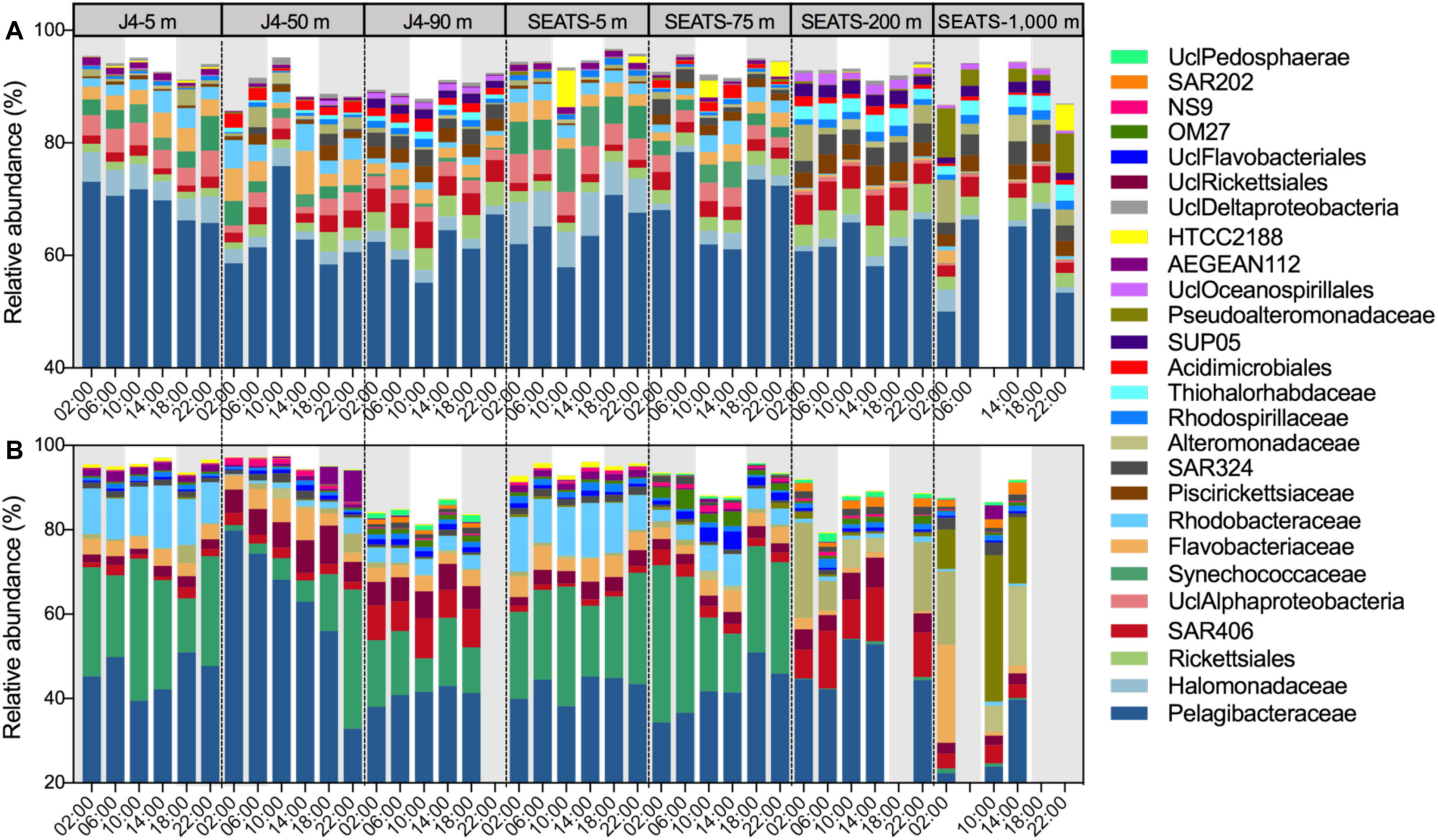
The diel cycling of the BCC in different layers of stations J4 and SEATS. The relative abundances of total BCC at the DNA level (A) and active BCC at the RNA level (B) at the family-level taxonomy. The top 20 bacterial families are given across all samples at each total and active BCC. Shaded areas represent the nighttime.

Sequence analysis based on the 16*S* ribosomal RNA (rRNA) gene and 16*S* rRNA (hereafter DNA community and RNA community, respectively) was used as proxies for the diversity and composition of the total and active bacterial communities (Figs. 3 and 4). Overall, the diel variability of the alpha diversity [assessed using the operational taxonomic unit (OTU) richness and the Shannon diversity index] of the RNA community showed distinctly more substantial variation than the DNA community at most detected locations (Fig. S2). At station J4, compared to the relatively invariant change at the bottom layer, higher alpha diversity values of both DNA and RNA communities were generally found at dawn and dusk in the surface and DCM layers [analysis of variance (ANOVA), *P* < 0.05]. At the SEATS station, a higher alpha diversity of the RNA community appeared at midnight in the surface layer but at noon in the DCM layer.

Nonmetric multidimensional scaling (NMDS) and clustering analyses based on Bray–Curtis similarity revealed that both the DNA and RNA communities formed 3 distinct groups (surface, subsurface, and mesopelagic) primarily concerning spatial distribution rather than diel periodicity (Fig. 3), similar to that found in a recent depth-resolved study over the diel cycle near Hawaii [26]. A one-way analysis of similarity also revealed that day–night samples from detected layers of J4 and SEATS stations were not marked different [permutational multivariate analysis of variance (PERMANOVA); *P* > 0.05]. Among the DNA and RNA communities, the population of Pelagibacter (SAR11 clade) bacteria (with average relative abundances of 64.5% and 44.6% in the DNA and RNA communities, respectively) dominated the community in all layers of J4 and the surface and DCM layers of SEATS, while Alteromonadaceae and Pseudoalteromonadaceae were more abundant in the boundary and mesopelagic layers of SEATS (Fig. 4). In the surface, DCM, and boundary layer of SEATS, SAR11 presented a pronounced diel variation within the DNA community with a higher abundance at night and a minimum around noon, which is consistent with the pattern revealed by previous finding [27].

### Contrasting diel patterns of viral dynamics in different environments

The viral abundance (VA) dynamics in surface water at both J4 and SEATS showed apparent diel variability with a peak at 10:00 (J4, 10.76 ± 0.01 × 10^6^ cells·ml^−1^; SEATS, 7.54 ± 0.13 × 10^6^ cells·ml^−1^), followed by lower abundances at mid-day (Fig. 1B and D). Such a diel trend was previously reported [18,28], although less pronounced than ours. A different diel oscillation with distinctly higher abundances recorded around noon occurred in the DCM and bottom layer of J4 as well as the DCM and boundary layer of SEATS (Fig. 1F, H, J, and L). In contrast, no pronounced pattern was found in the mesopelagic layer. In general, the diel oscillation of virus-to-bacteria ratio (VBR) followed the VA in most layers, except for higher values found at 22:00 in the surface and DCM layers of SEATS (Fig. 1D and H).

The lytic viral production (VP) in the surface water of J4 and SEATS also shared a pronounced nighttime-production pattern with lytic VP values at midnight (J4, 2.55 ± 0.39 × 10^5^ ml^−1^·h^−1^; SEATS, 2.29 ± 0.21 × 10^5^ ml^−1^·h^−1^) being approximately 7 times higher than lytic VP values at noon (Fig. 2B and D); a similar diel viral fluctuation was found in the surface water of the North Sea [18]. In the DCM layer at J4, the significantly lower values of lytic VP were found at 06:00 and 18:00 compared with other time points (Fig. 2F; ANOVA, *P* = 0.004), and no distinct diel variation was found in the bottom layer. A different daytime-production pattern was observed in the DCM and boundary layers of SEATS (Fig. 2H and L), with a maximum lytic VP value occurring at noon and lower values detected at nighttime. Lysogenic VP generally followed the diel variability of lytic VP in all detected layers of the SEATS station (Fig. 2D, H, L, and N). However, at station J4, especially in the surface and bottom layers, the diel fluctuation of lysogenic VP showed a different pattern from that of lytic VP, showing that the lysogenic VP also had a higher value around noon (Fig. 2B and J).

At station J4, there was no apparent diel pattern of viral decay (VD) in any layer (Fig. 2A, E, and I). In contrast, in the surface, DCM, and boundary layers of SEATS, a pronounced daytime-decay pattern with higher values of VD occurred around noon was recorded, and no such pattern was detected in the mesopelagic layer. Distance-based multivariate analysis for a linear model (DISTLM) analysis found that the diel dynamics of VD contributed well to the change in lysogenic VP in the boundary and mesopelagic layers of SEATS (Table S2).

### Viral contribution to contrasting bacterial diel dynamics

Overall, depth-independent but habitat-specific diel patterns of virus–host interactions were found in contrasting coastal and open ocean sites. In the surface layer of coastal J4 and open ocean of SEATS, the shared night-production diel pattern of lytic VP was concurrent with the lower VD at night (Fig. 2A to D), thus contributing to the accumulation of the viral population size during the night. In contrast, in the DCM and boundary layers of open ocean of SEATS, the co-occurrence of daytime-production and daytime-decay diel patterns for viruses was observed (Fig. 2G, H, K, and L). These contrasting diel patterns for viral activities varying with water depth were found tightly coupled with the diel cycling of the bacterial activity. Differing from the gradually diminishing peak value of BP during the daytime with increasing water depth, the increasingly enhanced lytic VP around noon appeared with increasing depth. These results indicated that the high BP around noon in the surface water appeared with the low lytic VP, but the distinctly higher lytic VP was synchronized with the lower BP around noon in the subsurface layer. This result revealed that the pressure of high lytic VP might depress the bacterial metabolic activity over the diel period.

**Fig. 5.**
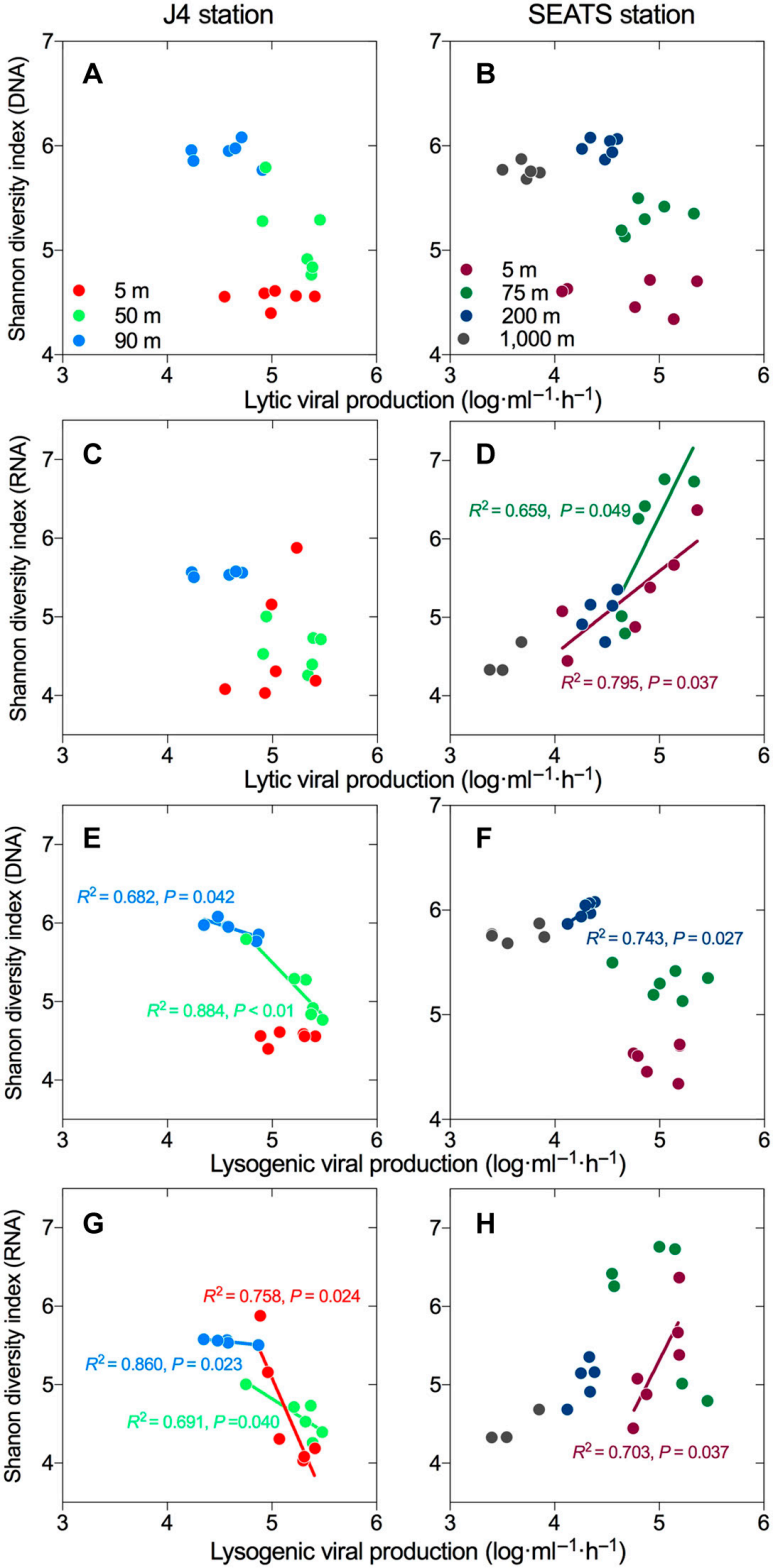
The linear relationship between VP and bacterial community diversity. The Shannon diversity index of the total bacterial community at the DNA level is plotted against the logarithmically transformed lytic VP at station J4 (A) and SEATS (B) and against the logarithmically transformed lysogenic VP at station J4 (E) and SEATS (F). The Shannon diversity index of the active bacterial community at the RNA level plotted against the logarithmically transformed lytic VP at station J4 (C) and SEATS (D) and against the logarithmically transformed lysogenic VP at station J4 (G) and SEATS (H). The lines with a significance level of *P* < 0.05 are given with the values of *R*^2^ and *P* on the graphs.

Interestingly, the different diel cycling of viral activities (i.e., lytic and lysogenic infection) also showed a contrasting coupling with the bacterial community diversity between coastal J4 and open ocean of SEATS. In the upper ocean of SEATS, a higher value of lytic VP (e.g., 2.29 ± 0.21 × 10^5^ ml^−1^·h^−1^ at 02:00 on the surface) was generally found with a higher bacterial community diversity (e.g., 6.37 of Shannon index of the RNA community at 02:00 on the surface). In fact, the Shannon diversity index of the RNA community in the surface and DCM layer of SEATS showed significantly positive correlations with the diel variation of lytic VP (Fig. 5D; linear regression, *P* < 0.05), indicating that stronger viral lysis contributed to higher community diversity. In contrast, at all layers of coastal J4, the diel variations of the Shannon index of DNA or RNA communities were found to be significantly and negatively correlated with lysogenic VP (Fig. 5E and G), showing that lower bacterial community diversity (e.g., 4.77 of Shannon diversity of the DNA community at 10:00 in the DCM) occurred at higher lysogeny values (e.g., 3.03 ± 0.96 × 10^5^ ml^−1^·h^−1^ at 10:00 in the DCM).

Beyond viral lysis and lysogeny contributing to contrasting diel patterns of community diversity, redundancy analysis showed that the diel variations in the total and active community compositions in the surface, subsurface, and mesopelagic groups all significantly corresponded to the changes in lytic and lysogenic VP (Fig. S3), suggesting that the diel dynamics of bacterial community was substantially affected by viral activity. DISTLM analysis also showed that in addition to the abundances of photoautotrophs, the diel dynamics of VP or VA significantly contributed to explaining the diel variability of the bacterial total and active community composition (Table S3), confirming the critical role of viruses in shaping bacterial diel dynamics. Taken together, our results revealed the tightly synchronized depth-dependent and habitat-specific diel patterns of virus–host interactions, suggesting that viruses can serve as a crucial top-down factor influencing the diel cycling of the bacterial community in the ocean.

## Discussion

Over the diel period, shifts in the bacterial community were previously found to be drastic and rhythmic, and their main drivers are generally assumed to be the periodic light intensity or coordinated coupling of photoautotrophs and heterotrophs [8,10,29,30]. However, it is largely unknown to what extent viral infection affects bacterial diel dynamics [21,31,32]. Here, our results revealed a distinct viral impact on the diel cycling of the bacterial community through depth-independent and habitat-specific virus–host interactions. The detected positive coupling between the bacterial community diversity and the diel change of the lytic VP in the open ocean is concurrent with the scenario that high viral lysis could diversify the bacterial community according to the “Kill-the-Winner” mode [15]. In this sense, during the diel cycle, the dominant populations, as potentially winning bacterioplankton for resource competition, according to the Kill-the-Winner hypothesis, are more likely lysed by lytic viruses [16]. A tight coupling of the abundances of SAR11 (one of the most dominant bacterioplankton in the ocean) and viruses was observed in our study (Figs. 1 and 4). Minimum abundances of SAR11 concurred with the maximum values of VA, thereby suggesting a predator–prey relationship and that viral lysis is a major control factor for the SAR11 population [33]. Viral predation is thought to stimulate species-level host diversity through lineage-specific predation, contributing to a more diverse bacterial community even at short-term time scales such as the diel cycle [17,34].

Conversely, in the coastal station, the bacterial community diversity was negatively coupled with the diel change of lysogenic VP, revealing another diel pattern of virus–host interaction in which high lysogeny would result in lower host diversity (Fig. 5C and D). This observation is potentially due to an alternative viral control mechanism, “Piggyback-the-Winner”, i.e., temperate viruses would use lysogenic infection to help their bacterial hosts survive during diel cycling [35]. Lysogenic bacterioplankton would dominate the bacterial community through enhanced competitive fitness or resistance to superinfection by relative lytic viruses, thus reducing the diversity among the bacterial community [17,36,37]. The Piggyback-the-Winner hypothesis suggested that lysogeny could play a more critical role in high-bacterioplankton-density environments [35,37]. Indeed, our results showed that diel changes in bacterial community diversity were tightly linked with lytic infection in the open ocean but with lysogenic infection in coastal habitats with higher bacterial abundance. These results indicate that viruses can not only kill but also piggyback the bacterial host, dependent on the selected viral strategies in specific environments, serving as a crucial top-down factor influencing the diel cycling of the bacterial community in the ocean [14,31].

The impact of predation versus rhythmic patterns on bacterial community composition is a fundamental topic in bacterial ecology that remains largely unanswered [38]. Significant linkages between viral activities and diel dynamics of different bacterial populations were found (Fig. S4), although their coupling showed a considerable degree of heterogeneity that varied with habitat, indicating the potential impact of viral predation on bacterial diel dynamics at the species level [39,40]. In particular, the diel dynamic pattern of the abundances of viruses and SAR11 bacteria showed a predator–prey relationship within the euphotic zone of oligotrophic open ocean. Consistent with a previous study, SAR11 bacterioplankton, a widely distributed and ecologically important clade in the ocean [41], exhibited pronounced diel oscillation in the open ocean but not in coastal seas. It was hypothesized that the phylogenetically related coastal and open ocean SAR11 populations might be under different controlling factors [9]. Compared to other bacterioplankton with larger genomes and corresponding increased metabolic and regulatory versatility, SAR11 harbors highly streamlined genomes with reduced regulatory machinery. Hence, SAR11 exhibit potentially weak metabolic dynamics in response to diel changes of bottom-up controlling factors but might be largely constrained by global transcriptional processes that are greatly affected by viral infection [29]. Indeed, abundant lytic viruses infecting SAR11 in the ocean have been characterized [33]. Recently, temperate SAR11 viruses were also isolated and found to be widely distributed across the ocean. Interestingly, it was found that temperate SAR11 viruses had contrasting virus–host interactions under different trophic conditions; they maintained more lysogeny under carbon-replete growth conditions but enhanced lysis in carbon-limiting growth ecosystems [42]. Given that the dominant species within communities was SAR11 bacteria (64.5% and 44.6% of the DNA and RNA communities, respectively), the differential viral strategies to infect SAR11 potentially contributed to the revealed diel cycling of virus–host interactions in which viral lysis was a major driver of bacterial community dynamics in the oligotrophic open ocean but lysogeny played a more important role in the coastal ocean [31].

Previously, a common diel trend of viral density during incubations for viral infection was reported, i.e., the development of the viral population was often remarkably similar concerning the time of day [18]. However, such a trend was not found in our results (Fig. S5), suggesting that the diel rhythm of viral infection might not have been constrained by a “time clock” scenario in which the release of viral progeny occurred mainly at a specific time of day. Moreover, the maximum abundance of viruses during incubations to detect viral infection or production was assumed to infer the major viral lysis event after by the general transcriptional process of the viral community. Hence, the time length to maximum abundance (TMA) was considered, to a certain extent, to indicate the viral infection capability, in which a shorter value of TMA generally suggests a more vital lytic ability and a faster release of virions [18,28]. We found that the TMA varied with sampling time points and environments (Fig. S6), suggesting that diel viral rhythm might not be limited to the time-delayed virion increase pattern controlled by the constant viral transcriptional process [20]. It has long been assumed that the viral lysis–lysogeny switch could occur over the diel cycle [18]. It was hypothesized that the viral community preferred lysogenic infection to integrate into the bacterial genome during the daytime to escape the direct damage to viral particles from strong sunlight, whereas the switch from lysogeny to lysis during the daytime could also occur because of the viral light-dependent life history traits, i.e., prophage induction by sunlight-induced host DNA damage or increased host activity [18,21,43]. In our results, no evident viral lysis–lysogeny switch was found over the diel cycle at the water column of either the coastal or open ocean.

Periodic environmental signals and subsequent bacterial responses shape marine biogeochemical cycles. Understanding viral impacts on the marine food web is vital for accurately modeling carbon fluxes in the ocean since mortality through the viral lysis of bacterioplankton would result in the release of relatively labile dissolved organic matter (DOM) to the marine DOM pool that fuels heterotrophic bacterial growth [12,44]. Moreover, it was considered that strong viral lysis coordinated to remove a substantial proportion of bacterial taxa over the diel period might largely influence the metabolic activity of the bacterial community [27,32]. On the diel scale, anticorrelated variation between viral lytic infection and bacterial metabolic activity was found in our study and previous investigations [18,28]. In the surface water of the open ocean, the lower lytic VP during the daytime indicates low viral lysis of heterotrophic bacterioplankton. Hence, bacterioplankton could effectively take up DOM released extracellularly during phytoplankton photosynthesis, which fuels bacterial metabolism and enhances energy transfer from the phototrophic population to the heterotrophs [12,45]. In the subsurface open ocean, in contrast, high viral lysis during the daytime would inhibit the bacterial uptake of labile DOM produced by phytoplankton, leading to the lessened bacterial metabolic activity observed during the daytime. Consequently, the carbon fixed by photosynthesis would be less channeled into the bacterial community during the daytime. Our results suggest a potentially important role of viruses in global carbon cycling over short-term dynamics [12,46]. Further work, especially in complex marine ecosystems with different time scales exposed to various environmental factors, to better explore virus–host interactions would narrow down the knowledge gap of the multiple viral roles in bacterial ecology and biogeochemical processes.

**Fig. 6.**
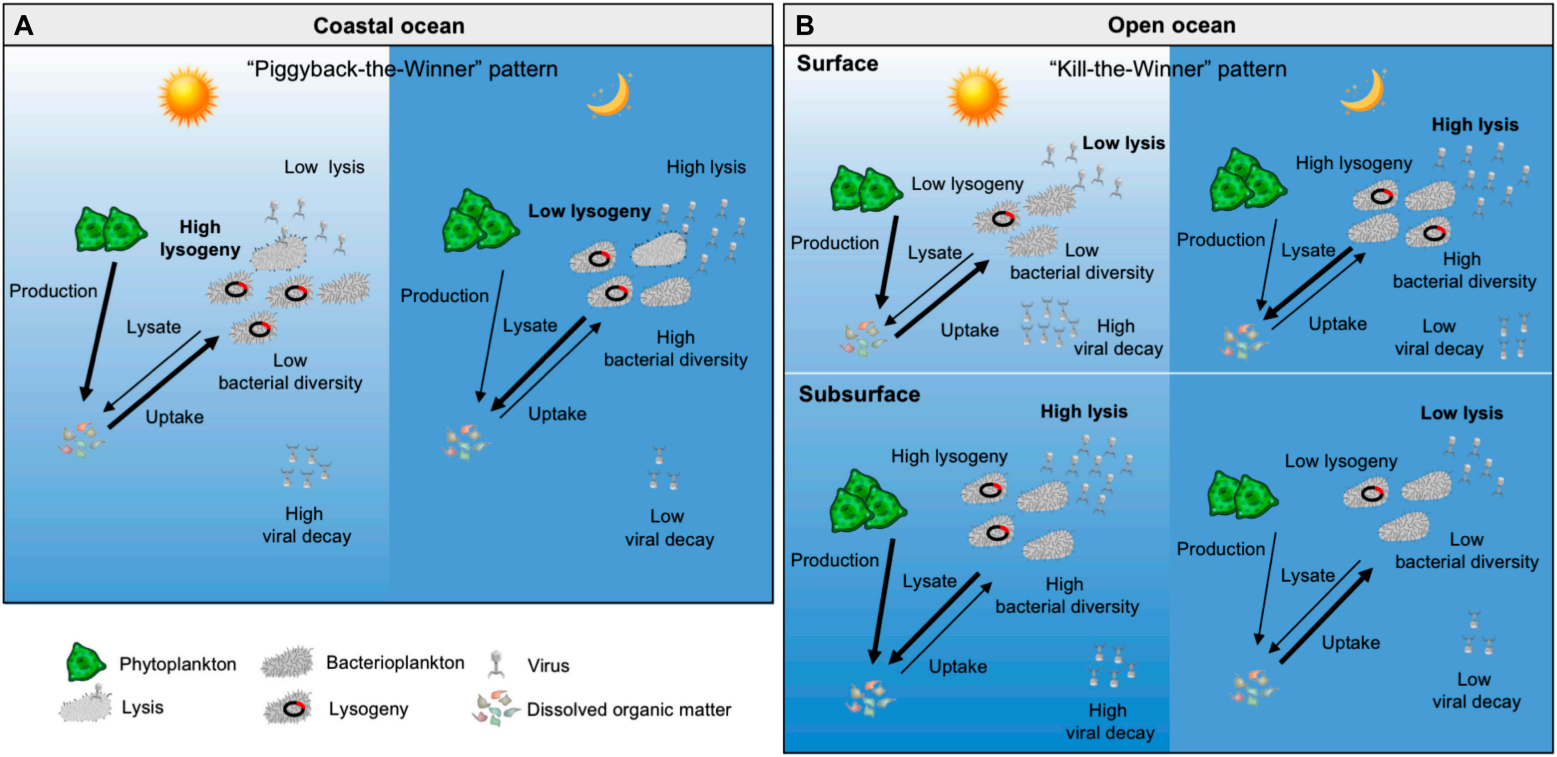
Schematic overview depicting the depth-independent and habitat-specific diel patterns of virus–host interactions. In the coastal ocean (A), low viral lysis but high VD is observed during the daytime, while the opposite pattern appears at the nighttime, leading to an accumulation of viral populations during the night. Moreover, high lysogeny is linked to low bacterial diversity in the costal ocean, following the scenario as suggested by “Piggyback-the-Winner” mode, which posits that a viral lysogenic lifestyle can potentially help their host dominate the bacterial community. In the open ocean (B), the diel pattern of viral lysis and decay at the surface water is similar to that of the coastal ocean, suggesting that bacterioplankton may effectively uptake primary produced DOM under conditions of reduced virus-mediated mortality. The virus–host interactions at the subsurface water show an opposite scenario, revealing that high viral lysis during the daytime potentially contribute to the production of DOM. In addition, high viral lysis is linked to high bacterial diversity at open ocean, indicating that viral lysis can diversify the bacterial community as suggested by “Kill-the-Winner” mode.

Taken together, this study presents a comprehensive assessment of viral dynamics and their impact on the bacterial community during diel cycling in coastal and open ocean water columns (as summarized in Fig. 6). Contrasting diel scenarios in viral and bacterial dynamics were found in contrasting marine environments, shaped by the depth-dependent and habitat-specific diel cycling of virus–host interactions impacted by viral lytic and lysogenic infection. The findings strongly indicate that viruses are an essential ecological driver of bacterial diel dynamics that can exert an intense influence on marine bacterial ecology. The revealed diel patterns of virus–host interactions in this study also potentially provided additional insights into the exploration of the viral impact on bacterial processes and biogeochemical functions in the ocean.

## Materials and Methods

### Study sites and sampling

The study was conducted at 2 stations (coastal versus open ocean) during a summer cruise aboard *R/V Shiyan I* to the SCS in August and September 2014 (Fig. S1). Coastal station J4 is located on the continental shelf (103 m in depth), which is exposed to higher coastal runoff influence, while the oligotrophic open ocean station SEATS is located further offshore with a depth of 3,846 m. Water in discrete depth layers of surface (5 m), DCM (50 m), and bottom (90 m) for station J4 and surface (5 m), DCM (75 m), the boundary of the euphotic zone (200 m), and mesopelagic (1,000 m) for station SEATS were collected using 10-liter Niskin bottles mounted on a conductivity–temperature–depth (CTD) rosette (SBE9/11 plus, Sea-Bird Electronics Inc., USA). The location of the DCM layer was determined by the fluorescent probe on the CTD rosette.

The sampling of diel cycle water samples followed the Eulerian time-series strategy that samples were taken at fixed geographic locations over time [47]. Given the complexities and challenges associated with field expedition research in the ocean and to better explore the virus–host interaction, samples were taken for as many viral and bacterial parameters as possible within a single diel cycle, rather than collecting a limited number of parameters across multiple diel cycles. Hence, to collect water samples at 6 time points in a diel cycle, a series of CTD casts were performed at 02:00 and 14:00, 06:00 and 18:00, and 10:00 and 22:00 (local time) on 25 to 27 August for the station SEATS and on 4 to 6 September for the station J4. The sunrise occurred ca. 06:00, and the sunset occurred ca. 18:00 (Fig. S1B). No rainfall or fog events were recorded at sites during the sampling. Corresponding temperature and salinity data were derived from the probe mounted on the CTD rosette. Samples for determining the Chl *a* concentration were collected by 0.7-μm glass fiber filters with fine porosity (GF/F filters) (47 mm; Whatman) and determined by a Turner Designs Fluorometer.

The samples for biological analysis were prefiltered through 20-μm mesh filters to remove large particles and zooplankton. For bacterial abundance and VA, 2 ml of subsamples were fixed with a final concentration of 0.5% glutaraldehyde at room temperature for 15 min in the dark. Two liters of water for each DNA and RNA analysis were filtered through 0.22-μm pore-size polycarbonate filters (47 mm; Millipore) at a pressure of <0.03 MPa. Samples for RNA analysis were collected within 30 min and stored in 2-ml RNase-free tubes with RNAlater solution (Ambion). All filters and abundance samples were stored at −80 °C for later analysis after flash-freezing in liquid nitrogen.

### Bacterial abundance and VA

HBA was determined after staining with SYBR Green I (Molecular Probe, USA), and Pro, Syn, and Peuk were counted directly without staining by the flow cytometry (Epics Altra II, Beckman Coulter, USA) according to the established protocol [48]. The VA was measured using the same flow cytometer after SYBR Green I staining. The VBR was calculated by dividing VA with HBA. All data for flow cytometric analysis were analyzed with FlowJo v10.6 software (Becton, Dickinson & Company, USA).

### Bacterial production

The BP was determined by ^3^H-leucine incorporation following the method previously described with some modifications [49]. Briefly, triplicate 1.5-ml aliquots of water samples and one trichloroacetic acid (TCA)-killed control (1% final concentration) were incubated with ^3^H-leucine (16 Ci·mmol^−1^) to a saturating concentration of 20 nM for 2 h of incubation in the dark at in situ temperature. After adding 5% TCA (final concentration) and centrifuging the sample at 16,000*g* for 10 min, the supernatant was extracted twice with 5% ice-cold TCA and rinsed with 80% ethanol. The radioactivity was measured with a liquid scintillation counter (280TR, Waltham, USA), and the rate of incorporation of ^3^H-leucine was calculated as the average rate of the triplicate leucine incorporation rates corrected by the control.

### VP rate

The VP was estimated by the viral reduction approach [50], following the procedure previously described [46]. Briefly, an approximately 600-ml water sample was filtered by a tangential flow filtration (TFF) with a 0.2-μm pore-size cartridge (Labscale, Millipore, USA) to obtain a 50-ml bacterial concentrate and a ca. 500-ml filtrate. The filtrate was then TFF filtered with a 30-kDa pore-size cartridge (Millipore) to generate virus-free water. Fifty milliliters of bacterial concentrate was diluted and gently mixed with 250 ml of virus-free water, and then 200 ml of the mixture was aliquoted into four 50-ml sterile centrifuge tubes to incubate at in situ temperature in the dark. Two sets of incubation experiments were performed simultaneously. One set was taken as control to measure the lytic VP. The other set was the same as the control but with mitomycin C added (final concentration of 1 μg ml^−1^; Sigma-Aldrich), which could introduce temperate virus into the lytic cycle. Replicate subsamples of 1 ml for viral and bacterial enumeration were taken at 3-h intervals during the 15-h incubation. With the help of the online program VIPCAL [51], we calculated the lytic VP from the control group and lysogenic VP from mitomycin C addition group. The VA in each incubation for the lytic VP was normalized to a mean of zero and unity variance to compensate for the absolute differences across incubations in contrast sampling time and locations (Fig. S5) [18]. The time span (TMA) between *T*_0h_ and the time when the highest observed VA occurred within these VP incubations was calculated and serves as an index of the short-time-scale viral replication cycle as previously described (Fig. S6) [28].

### VD rate

The VD rate was measured according to Noble and Fuhrman [52]. Seawater was filtered by TFF with a 0.22-μm-pore-size cartridge to remove bacterioplankton and particles larger than 0.22 μm. The 50-ml filtrate in replicates was incubated at in situ temperature in the dark. Replicate subsamples of 1 ml were collected for bacterial and viral enumeration at 3-h intervals up to 15 h. The VD was calculated as the slope of the linear regression fitted to the natural logarithm of VA plotted against time during 15 h of incubation.

### DNA and RNA extraction and sequencing

Bacterial DNA was extracted using a PowerSoil DNA Isolation Kit (MoBio Laboratories, USA) following the manufacturer’s protocols. RNA samples were extracted and reverse-transcribed into cDNA as previously described [53]. The V3–V4 region of the bacterial 16*S* rRNA gene in each DNA and cDNA sample was amplified using the specific primers 341F (CCTAYGGGRBGCASCAG) and 806R (GGACTACNNGGGTATCTAAT) complemented with sample-specific barcodes. Nucleic acid paired-end sequencing (2 × 250) was performed on an Illumina MiSeq platform following the standard protocol.

### Bacterial community composition

Libraries of sequences and OTUs were analyzed by MOTHUR (v. 1.36.1) following the standard operating procedure (https://www.mothur.org/wiki/MiSeq_SOP) [54]. Briefly, sequences that contained more than one ambiguous nucleotide did not have a complete barcode and primer at one end, or sequences that were shorter than 200 bp after removal of the barcode and primer sequences were eliminated. Chimera were also identified and removed. For OTU classification, reads were clustered at 97% similarity, and taxonomy was assigned using the SILVA ribosomal database (Release119, http://www.arb-silva.de) [54,55]. All chloroplast and mitochondrial sequences were removed from the dataset. The richness (OTUs) and diversity (Shannon diversity index) estimates of the bacterial community were calculated on the basis of OTU assignment.

### Statistical analysis

Data normality was tested before analysis using Shapiro–Wilk *W* tests, and the data were logarithmically transformed to meet normality if necessary. ANOVA was used to determine the significant differences between samples. If a significant difference was observed, post hoc Tukey’s test was also performed. Linear regression was used to characterize the relationship between abiotic and biotic factors in GraphPad Prism 7 (GraphPad, USA). Redundancy analysis was performed to further analyze variations in the bacterial community composition under the constraint of abiotic and biotic variables after normalizing the data using CANOCO 4.5 software (Microcomputer Power, USA). DISTLM analysis was applied by Primer 6 with the PERMANOVA+ package (Primer-E, Plymouth, UK) to test the relationships between selected parameters or bacterial community composition and other parameters. NMDS and cluster analyses were also used to determine the similarity or grouping of samples to each other. All the differences were considered significant at *P* < 0.05.

## Acknowledgments

We wish to thank J. Wang, J. Sun, and M. He for assistance with the sampling and data analysis. We also thank the sampling support from the Senior User Project of RV KEXUE (KEXUE2020G10) of Center for Ocean Mega-Science, Chinese Academy of Sciences. **Funding:** This work was supported by the National Natural Science Foundation of China (41861144018, 42188102, 91951209, 41906085, and 2021YFE0193000). X.C. was supported by the PhD Fellowship of the State Key Laboratory of Marine Environmental Science at Xiamen University. M.G.W. was supported by the international collaborative project on “Marine Biogeochemistry and Ecotoxicology” funded by the Program of Introducing Talents of Discipline to Universities (BP0719030). C.H. was supported by the China Postdoctoral Science Foundation (2021M691863) and the Natural Science Foundation of Fujian Province of China (2023J05017). Y.Y. was supported by China Postdoctoral Science Foundation (2019M662237). **Author contributions:** R.Z. and N.J. designed and coordinated the study. X.C., C.H., W.W., Y.Y., and H.L. performed the experiments. X.C., C.H., and R.Z. analyzed the data and wrote the manuscript with contributions from all coauthors. All authors contributed to the dataset, discussed the results, and suggested improvements to the manuscript. **Competing interests:** The authors declare that they have no competing interests.

## Data Availability

Data supporting the findings of this study are available in this manuscript and the Supplementary Materials. The DNA and RNA sequences reported have been deposited in the NCBI Sequence Archive under BioProject number PRJNA704766.

## Supplementary Materials

Supplementary 1 Figs. S1 to S6
Tables S1 to S3
