# Supplementary material for: Virus–Host Interactions Drive Contrasting Bacterial Diel Dynamics in the Ocean
Source: Research (Wash D C). 2023 Aug 22;6:0213. doi: 10.34133/research.0213 (PMC10443526; doi:10.34133/research.0213)
Supplement: Supplementary 1 — Figs. S1 to S6 Tables S1 to S3 [file research.0213.f1.docx]

Supplementary Materials


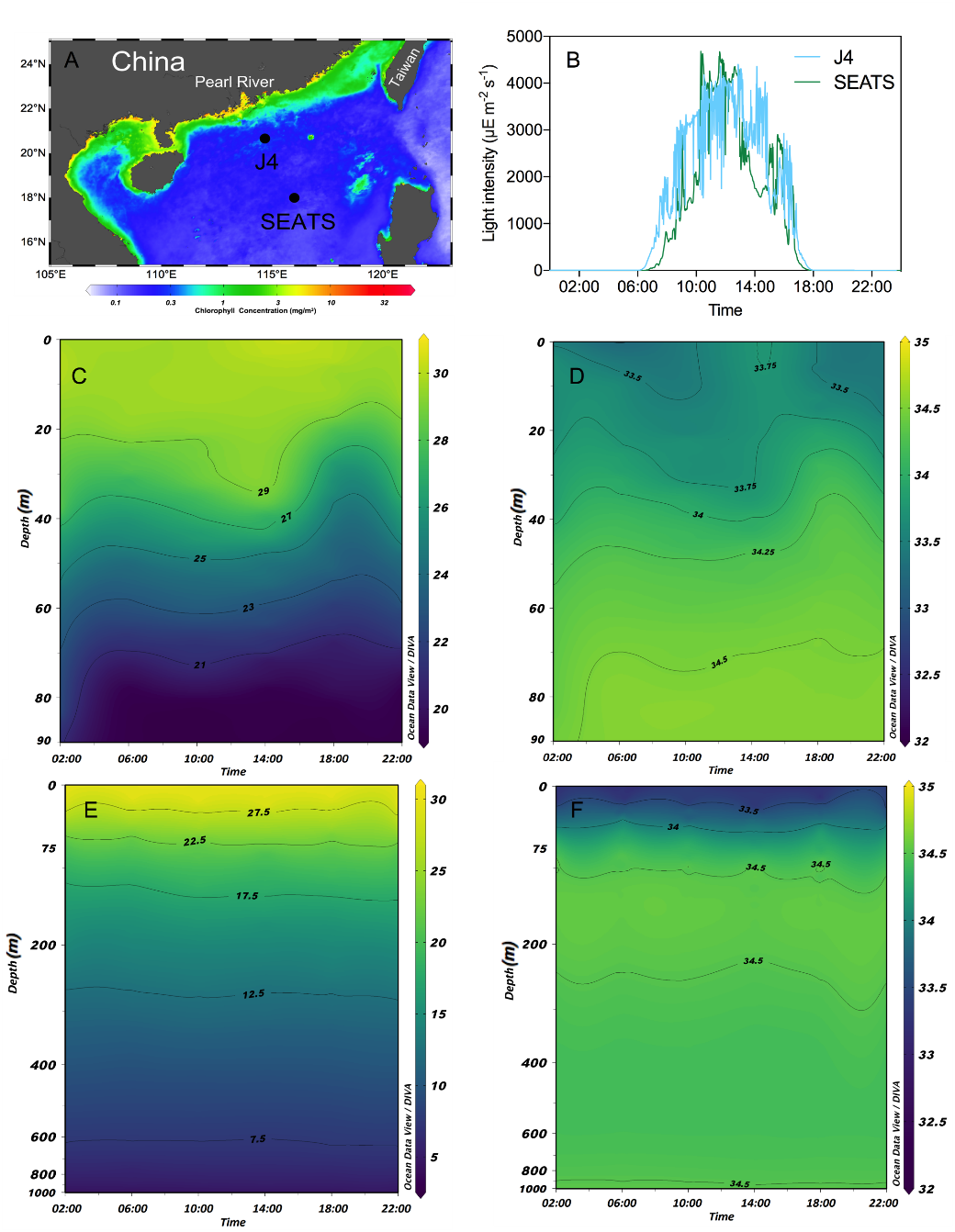


Fig. S1. Stations and sampling features in the South China Sea (SCS). (A) At the sampling sites in the SCS, coastal station J4 is located on the continental shelf, and open ocean station SEATS is in the middle of the SCS basin. (B) The surface photosynthetically available radiation (PAR) of stations J4 and SEATS during sampling. The depth profile of temperature (C) and salinity (D) during the sampling period at station J4 and the temperature (E) and salinity (E) during the sampling period at station SEATS.


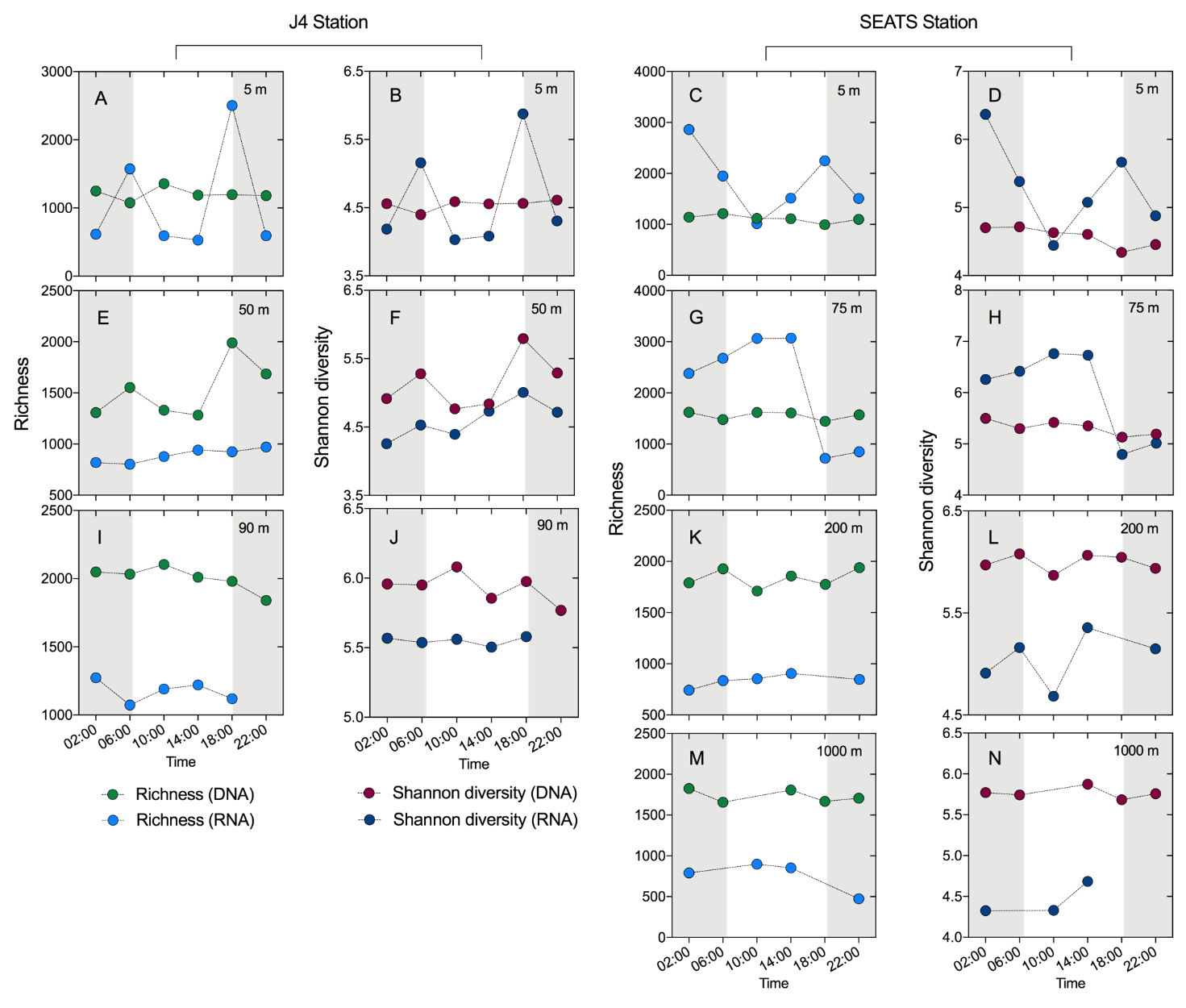


Fig. S2. Diel variability in the richness and diversity of the bacterial community. The index of OTU richness based on DNA and RNA levels in the 5 m (A), 50 m (E) and 90 m (I) layers of station J4 and in the 5 m (C), 75 m (G), 200 m (K) and 1000 m (M) layers of station SEATS. The Shannon diversity index based on DNA or RNA level in the 5 m (B), 50 m (F) and 90 m (J) layers of station J4 and in the 5 m (D), 75 m (H), 200 m (L) and 1000 m (N) layers of station SEATS.


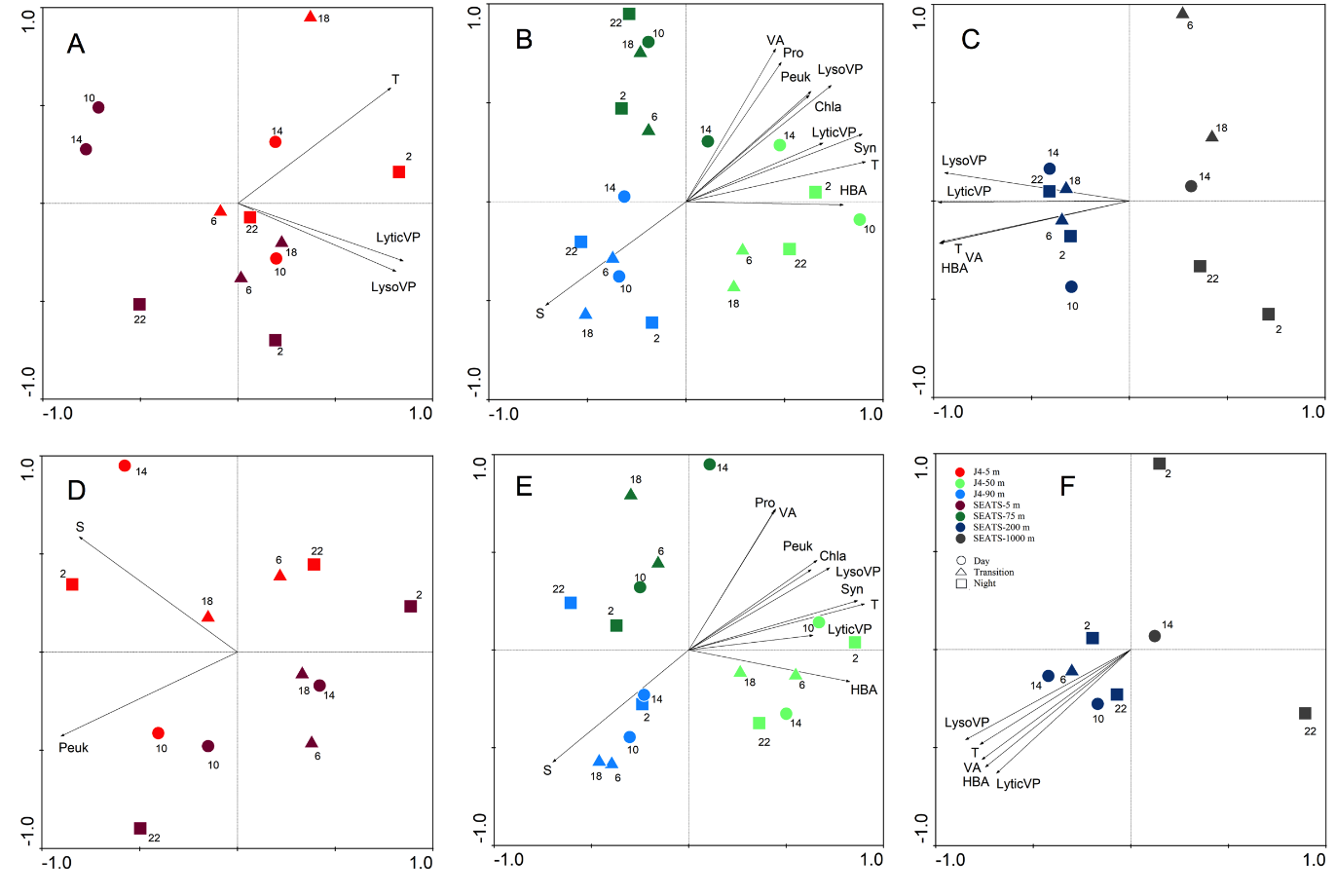


Fig. S3. Relationship between the bacterial community composition and environmental or biotic parameters. Redundancy analysis correlation (RDA) ordination describes environmental or biotic variables (black arrows, *P* < 0.05) that significantly explained the diel variability in bacterial community composition based on DNA level at the surface (A), subsurface (B) and mesopelagic (C) groups and based on the RNA level at the surface (D), subsurface (E) and mesopelagic (F) groups. The direction of the vectors indicates the effect of each driver variable on the two axes. The length of the arrow is proportional to the rate of change, whereas the direction represents the correlations to the axes. The different numbers of symbols represent the sampling time of the day and the different symbol shapes show the diel cycle of sampling. T, temperature; S, salinity; VA, viral abundance; HBA, heterotrophic bacterial abundance; VBR, virus-to-bacteria ratio; Pro, *Prochlorococcus* abundance; Syn, *Synechococcus* abundance; Peuk, picoeukaryotic abundance; BP, bacterial production; VD, viral decay rate and VP, viral production.


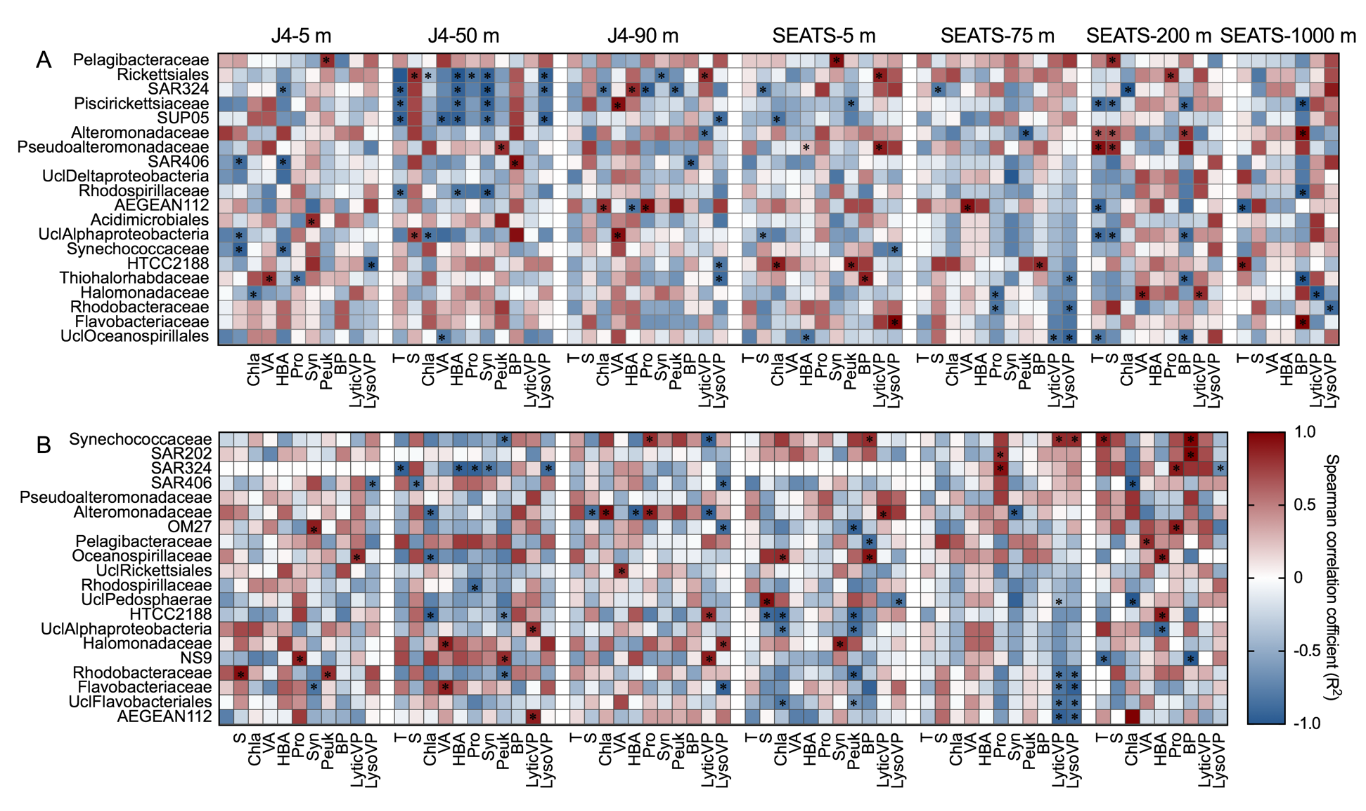


Fig. S4. Heat maps showing Spearman's rank correlation coefficient between the relative abundance of the bacterial community at the family level and abiotic or biotic parameters based on the DNA (A) and RNA (B) levels. The asterisk symbols represent the significance level (*P* < 0.05). T, temperature; S, salinity; Chl *a*, chlorophyll *a* concentration; VA, viral abundance; HBA, heterotrophic bacterial abundance; Pro, *Prochlorococcus* abundance; Syn, *Synechococcus* abundance; Peuk, picoeukaryotic abundance; BP, bacterial production and VP, viral production.


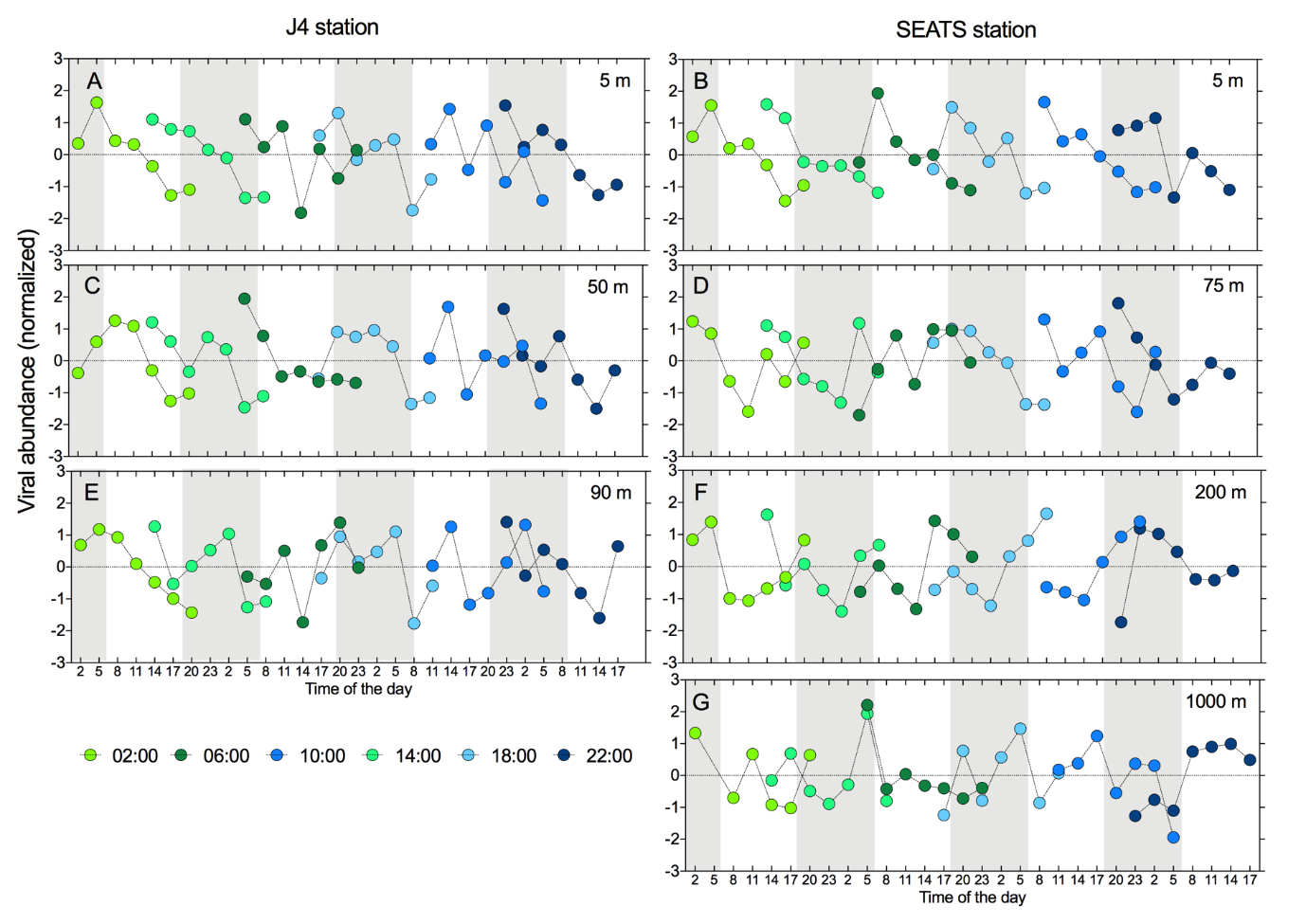


Fig. S5. Viral abundance over time during incubation to measure lytic viral production in different layers of station J4 and SEATS. Normalized average values of duplicate incubations are plotted against the sampling time in coordinated universal time (UTC). The different symbol colors represent the difference between the sampling time and the start of incubations. Shaded areas represent dark periods; error bars are not shown for clarity.


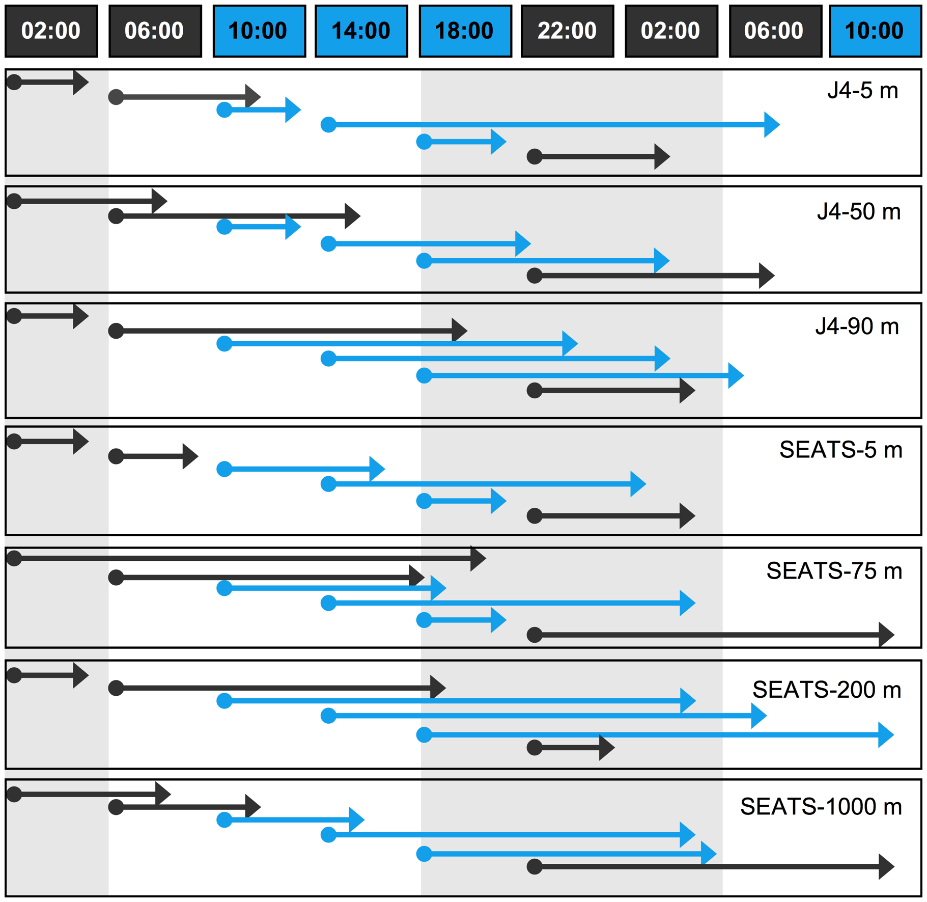


Fig. S6. A summary of lysis-dependent viral density increases during lytic viral production incubation is presented in Fig. S5. The viral density increases are assumed to be dependent only on the lysis event, and the time to maximum viral abundance within incubations hereby refers to the main viral lysis event. Colors of sampling times and arrows represent light-dark sample groups (blue: daytime, black: nighttime). The tails (indicated by circles) indicate the beginning of incubation, and the heads (indicated by triangles) of arrows indicate the time-point of viral density to the highest values. Shaded areas represent the dark period.

Table S1. Diel mean values of environmental and bacterial characteristics at the stations of station J4 and SEATS. The standard deviation (SD) is given after the mean value.

| **Parameters** | **Station J4** | | |  | **Station SEATS** | | | |
| --- | --- | --- | --- | --- | --- | --- | --- | --- |
|  | **5 m** | **50 m** | **90 m** |  | **5 m** | **75 m** | **200 m** | **1000 m** |
| Temperature (°C) | 29.84 (0.17) | 24.39 (0.64) | 19.29 (0.38) |  | 29.59 (0.11) | 21.42 (0.62) | 14.71 (0.18) | 4.62 (0.09) |
| Salinity | 33.54 (0.13) | 34.29 (0.06) | 34.54 (0.03) |  | 33.33 (0.06) | 34.34 (0.07) | 34.53 (0.01) | 34.52 (0.01) |
| NO_2_^-^ (μmol L^-1^) | 0.01 | 0.16 | 0.04 |  | / | 0.25 | / | / |
| NO_3_^-^ (μmol L^-1^) | 0.08 | 3.21 | 9.89 |  | 0.13 | 2.23 | 18.77 | 36.29 |
| PO_4_^3-^ (μmol L^-1^) | 0.17 | 0.42 | 0.82 |  | 0.2 | 0.42 | 1.26 | 2.3 |
| Si_3_^2-^ (μmol L^-1^) | 0.25 | 3.96 | 11.39 |  | 0.75 | 3.64 | 22.2 | 106.98 |
| Chl *a* (μg L^-1^) | 0.145 (0.007) | 0.606 (0.123) | 0.210 (0.081) |  | 0.139 (0.043) | 0.504 (0.118) | 0.008 (0.002) | / |
| VA (10^6^ mL^-1^) | 8.71 (1.40) | 7.63 (1.85) | 2.87 (0.64) |  | 6.54 (0.66) | 9.17 (2.31) | 1.76 (0.12) | 0.59 (0.06) |
| HBA (10^5^ cells mL^-1^) | 9.08 (0.28) | 7.98 (1.20) | 5.90 (0.30) |  | 9.18 (1.06) | 5.71 (1.42) | 1.97 (0.08) | 0.69 (0.10) |
| VBR | 9.58 (1.59) | 9.51 (1.70) | 4.86 (1.04) |  | 7.17 (0.71) | 16.28 (3.06) | 8.96 (0.38) | 8.56 (0.78) |
| Pro (10^4^ cells mL^-1^) | 0.03 (0.02) | 8.74 (2.33) | 1.76 (0.38) |  | 0.04 (0.02) | 11.22 (3.69) | 0.05 (0.02) | / |
| Syn (10^4^ cells mL^-1^) | 3.16 (2.55) | 6.84 (4.44) | 0.54 (0.22) |  | 10.53 (4.10) | 1.80 (1.24) | / | / |
| Peuk (10^3^ cells mL^-1^) | 0.69 (0.14) | 8.26 (5.17) | 1.16 (0.41) |  | 0.65 (0.18) | 5.1 (1.84) | / | / |
| Bacterial production (μg L^-1^ d^-1^) | 2.04 (1.94) | 2.14 (0.29) | 0.58 (0.31) |  | 2.24 (2.51) | 1.68 (1.37) | 0.24 (0.21) | 0.12 (0.06) |
| VD (%h^-1^) | 1.57 (0.66) | 2.17 (0.92) | 1.25 (0.35) |  | 2.38 (0.74) | 1.63 (0.88) | 1.53 (0.28) | 0.74 (0.15) |
| Lytic VP (10^5^ mL^-1^ h^-1^) | 1.25 (0.77) | 1.93 (0.86) | 0.42 (0.24) |  | 0.89 (0.83) | 0.92 (0.64) | 0.30 (0.08) | 0.05 (0.02) |
| Lysogenic VP (10^5^ mL^-1^ h^-1^) | 1.58 (0.72) | 2.02 (0.85) | 0.45 (0.22) |  | 1.10 (0.49) | 1.36 (0.87) | 0.30 (0.08) | 0.05 (0.02) |
| *Chl a, Chlorophyll a; VA, viral abundance; HBA, heterotrophic bacterial abundance; VBR, virus-to-bacteria ratio; Syn, Synechococcus abundance, Peuk, picoeukaryotic abundance; VD, viral decay; VP, viral production. The standard deviation is given after the mean value. "/" for not detection.* | | | | | | | | |
|  |  |  |  |  |  |  |  |  |

Table S2. Results of the multivariate regression analysis with forward selection (DISTLM forward) to explain the variability in the abundance of virus (VA) and heterotrophic bacteria (HBA), viral decay (VD), lytic viral production (Lytic VP) and lysogenic viral production (Lyso VP) in different layers of stations J4 and SEATS. Only results with significant level (*P* < 0.05) are shown. Pro, Syn and Peuk present the abundance of *Prochlorococcus*, *Synechococcus* and picoeukaryote; Chl *a*, Chlorophyll *a* concentration and BP, bacterial production.

| **Station-depth** | **Response variable** | **Selected variables** | **Pseudo-F** | ***R^2^*** | ***P*** | **Cumulative** |  | **Station-depth** | **Response variable** | **Selected variables** | **Pseudo-F** | ***R^2^*** | ***P*** | **Cumulative** |
| --- | --- | --- | --- | --- | --- | --- | --- | --- | --- | --- | --- | --- | --- | --- |
| J4-5 m | VA | Lytic VP | 8015.8 | 0.022 | 0.006 | 0.022 |  | SEATS-5 m | VD | Chl *a* | 2.049 | 0.339 | 0.027 | 0.339 |
|  | HBA | Peuk | 17290 | 0.093 | 0.001 | 0.094 |  |  | Lyso VP | Lytic VP | 20.067 | 0.834 | 0.015 | 0.834 |
|  | Lytic VP | Chl *a* | 7.753 | 0.660 | 0.032 | 0.660 |  |  |  | Chl *a* | 121.84 | 0.011 | 0.047 | 0.845 |
| J4-50 m | VA | Lyso VP | 21.347 | 0.842 | 0.011 | 0.842 |  | SEATS-75 m | VA | Syn | 11.061 | 0.734 | 0.031 | 0.734 |
|  | HBA | Syn | 31.8 | 0.888 | 0.008 | 0.888 |  |  |  | VD | 18.958 | 0.216 | 0.036 | 0.950 |
|  |  | Chl *a* | 16.99 | 0.025 | 0.035 | 0.913 |  |  | HBA | VD | 37.509 | 0.904 | 0.032 | 0.904 |
|  | Lytic VP | HBA | 8.746 | 0.264 | 0.040 | 0.263 |  |  |  | Lyso VP | 15.128 | 0.080 | 0.034 | 0.984 |
|  |  | Peuk | 97.615 | 0.009 | 0.044 | 0.344 |  |  |  | VA | 69.781 | 0.016 | 0.023 | 0.999 |
|  | Lyso VP | VA | 19.986 | 0.833 | 0.013 | 0.833 |  |  | VD | HBA | 24.022 | 0.857 | 0.032 | 0.857 |
|  |  | Pro | 21.57 | 0.146 | 0.021 | 0.980 |  |  | Lytic VP | T | 238.23 | 0.042 | 0.047 | 0.042 |
| J4-90 m | HBA | Chl *a* | 112.34 | 0.966 | 0.001 | 0.966 |  |  | Lyso VP | Syn | 27.62 | 0.873 | 0.010 | 0.873 |
|  | VD | BP | 25.199 | 0.372 | 0.030 | 0.372 |  |  |  | Pro | 323.61 | 0.047 | 0.015 | 0.920 |
|  | Lytic VP | Peuk | 17.373 | 0.813 | 0.030 | 0.813 |  | SEATS-200 m | VA | Lytic VP | 12.133 | 0.752 | 0.022 | 0.752 |
|  |  |  |  |  |  |  |  |  | HBA | VA | 7.869 | 0.663 | 0.0498 | 0.663 |
|  |  |  |  |  |  |  |  |  | Lytic VP | Pro | 23.01 | 0.852 | 0.006 | 0.852 |
|  |  |  |  |  |  |  |  |  | Lyso VP | VD | 15.949 | 0.799 | 0.017 | 0.799 |
|  |  |  |  |  |  |  |  | SEATS-1000 m | Lyso VP | VD | 6316.2 | 0.581 | 0.003 | 0.581 |

Table S3. Results of the multivariate regression analysis with forward selection (DISTLM forward) to explain the variability in the total (DNA) and active (RNA) bacterial community composition (BCC) in different layers of station J4 and SEATS. Only results with significant levels (*P* < 0.05) are shown. VP, viral production; VA, viral abundance; Syn, *Synechococcus* abundance; Peuk, picoeukaryote abundance, and T, temperature.

| **Station-depth** | **BCC** | **Selected variables** | **Pseudo-F** | ***R^2^*** | ***P*** | **Cumulative** |
| --- | --- | --- | --- | --- | --- | --- |
| J4-5 m | RNA | Lysogenic VP | 2.041 | 0.338 | 0.004 | 0.338 |
| J4-50 m | DNA | Syn | 2.903 | 0.421 | 0.004 | 0.421 |
|  | RNA | VA | 2.893 | 0.420 | 0.047 | 0.420 |
| J4-90 m | DNA | VA | 1.373 | 0.256 | 0.01 | 0.256 |
|  | RNA | Lytic VP | 1.501 | 0.334 | 0.009 | 0.334 |
| SEATS-5 m | DNA | Lysogenic VP | 3.434 | 0.462 | 0.004 | 0.462 |
|  | RNA | Peuk | 1.649 | 0.292 | 0.048 | 0.292 |
| SEATS-75 m | DNA | Syn | 3.667 | 0.478 | 0.005 | 0.478 |
|  | RNA | VA | 2.096 | 0.344 | 0.007 | 0.344 |
| SEATS-200 m | RNA | T | 3.104 | 0.509 | 0.023 | 0.509 |
